# Antiepileptic drug optimization using ambulatory electrocorticographic data from an implanted brain‐responsive neurostimulator

**DOI:** 10.1002/ccr3.2689

**Published:** 2020-01-30

**Authors:** Elysia Tjong, Yen‐Yi Peng

**Affiliations:** ^1^ A.T. Still University School of Osteopathic Medicine in Arizona Mesa AZ USA; ^2^ Amity Neurology Reno NV USA

**Keywords:** antiepileptic drug, electrocorticography, responsive neurostimulation system

## Abstract

Effectiveness of antiepileptic drugs (AEDs) has been traditionally monitored by patient‐reported seizures. A 31‐year‐old male patient was treated with a responsive neurostimulation system (RNS). The ambulatory electrocorticographic data were used to guide AED choice and dosing in achieving subsidence of disabling seizures and minimization of the adverse effects of polypharmacy.

## Graphical abstract

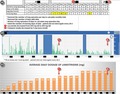

## 1 INTRODUCTION

The RNS^®^ System (NeuroPace) is a chronically implanted responsive neurostimulation system used for the adjunctive treatment of medically refractory partial onset seizures arising from ≤2 seizure foci. The neurostimulator is placed within the cranium, where it is connected to 2 four‐contact depth or cortical strip leads placed intracranially at the seizure onset zones. Each lead contains 4 sensing and stimulating electrodes.^1^, ^2^ The RNS System detects specific patterns of ECoG activity, provides closed‐loop electrical stimulation at a seizure focus, and stores ECoG activity, which are all based on physician‐programmed parameters.^1^, ^3^ Patient's data are transmitted to a secure online database (Patient Data Management System—PDMS), where they are available for physician review.^4^

“Detection” and “Long Episode” are two neurostimulator hourly count data on PDMS. The Detection events consist of times that the neurostimulator detects activity prespecified by the physician as abnormal. The Long Episode events consist of times that detection of abnormal activity continues beyond a preset physician‐defined duration.^3^ Other data include “Saturation” and “Swiping the Magnet”/“Magnet.” Saturation denotes events with amplitudes exceeding amplifier sensitivity, whereas Swiping the Magnet can trigger ECoG storage when the patient experiences any focal seizures. Approximately four 90‐seconds ECoG samples are stored at any one time, after which new ECoG recordings overwrite the oldest. To free neurostimulator memory, patients are instructed to transfer data daily and after any clinical seizure from the neurostimulator to PDMS. Thus, the numbers of ECoG recordings vary with detection parameters, the nature of the EEG pattern, and the frequency of which patients transfer the data.^3^ Therefore, the aforementioned counts of ECoG parameters do not always reflect the effectiveness of epilepsy control, especially in the beginning stages of RNS active programming.

**Figure 1:**
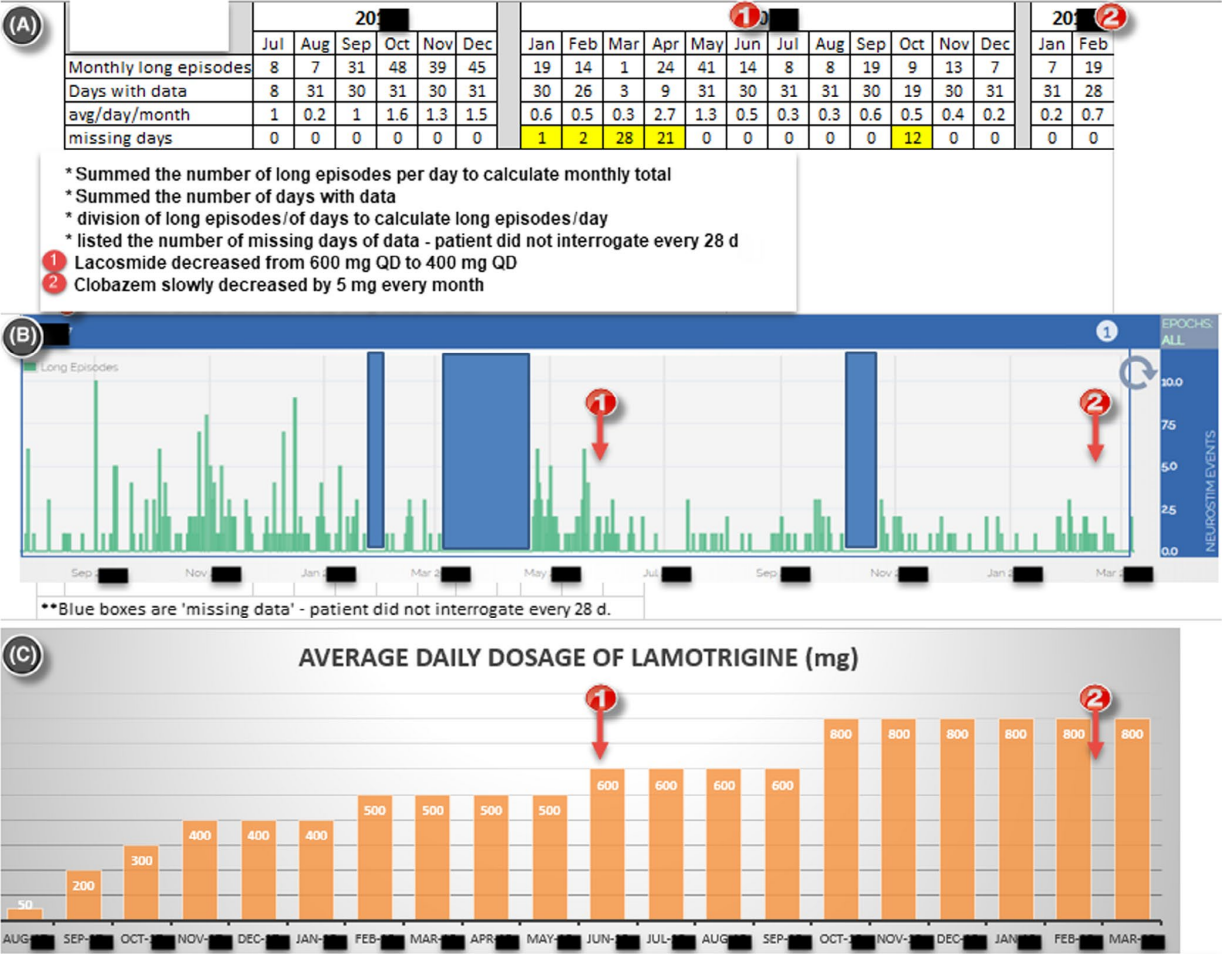
The Long Episode count showed a decreasing trend with a stepwise increase in Lamotrigine within a period of 20 mo. A, The monthly Long Episode count showed a decreasing trend. The Long Episode count is an overestimation of subclinical and clinical seizures because it also includes prolonged epileptiform and nondescript patterns that are not seizures. Lacosamide was reduced from 600 mg QD to 400 mg QD at the time of red circle one, and Clobazam was reduced at the time of red circle two. B, The monthly Long Episode count in the bar chart showed a decreasing trend within a period of 20 mo. Blue boxes represented the missing data since the patient did not upload the ECoG data every 28 d. C, The bar chart of the stepwise increase in Lamotrigine within a period of 20 mo

Despite these limitations, the Long Episode count of RNS could serve as a clinical marker that estimates both clinical and subclinical seizures with the assumption of both stable RNS programming parameters and reliable upload of ECoG data to the database (Figure 1). The Magnet count could also potentially serve as a marker of clinical focal seizures, but similarly, the patient must transfer the ECoG data to the secure online database daily and after any Magnet Swipe to avoid overwriting.

**Figure 2:**
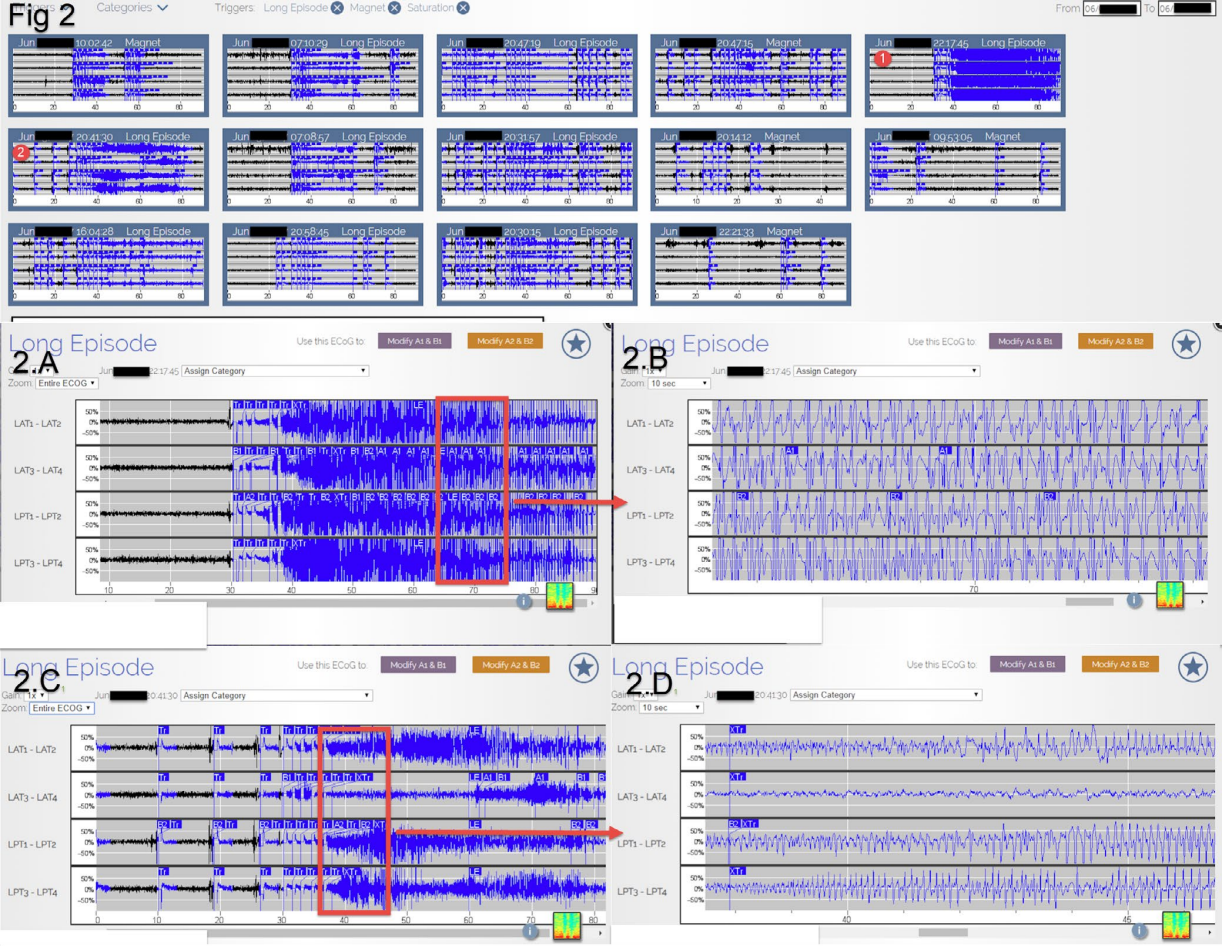
Three‐week period of recorded ECoG in the secure online database 1 month before the initiation of Lamotrigine (these ECoGs include Long Episode, Saturation, and Magnet). A is the enlargement of the red circle one, and B is the further enlargement of A and is consistent with an electrographic seizure. C is the enlargement of the red circle two, and D is the further enlargement of C and is consistent with an electrographic seizure

**Figure 3:**
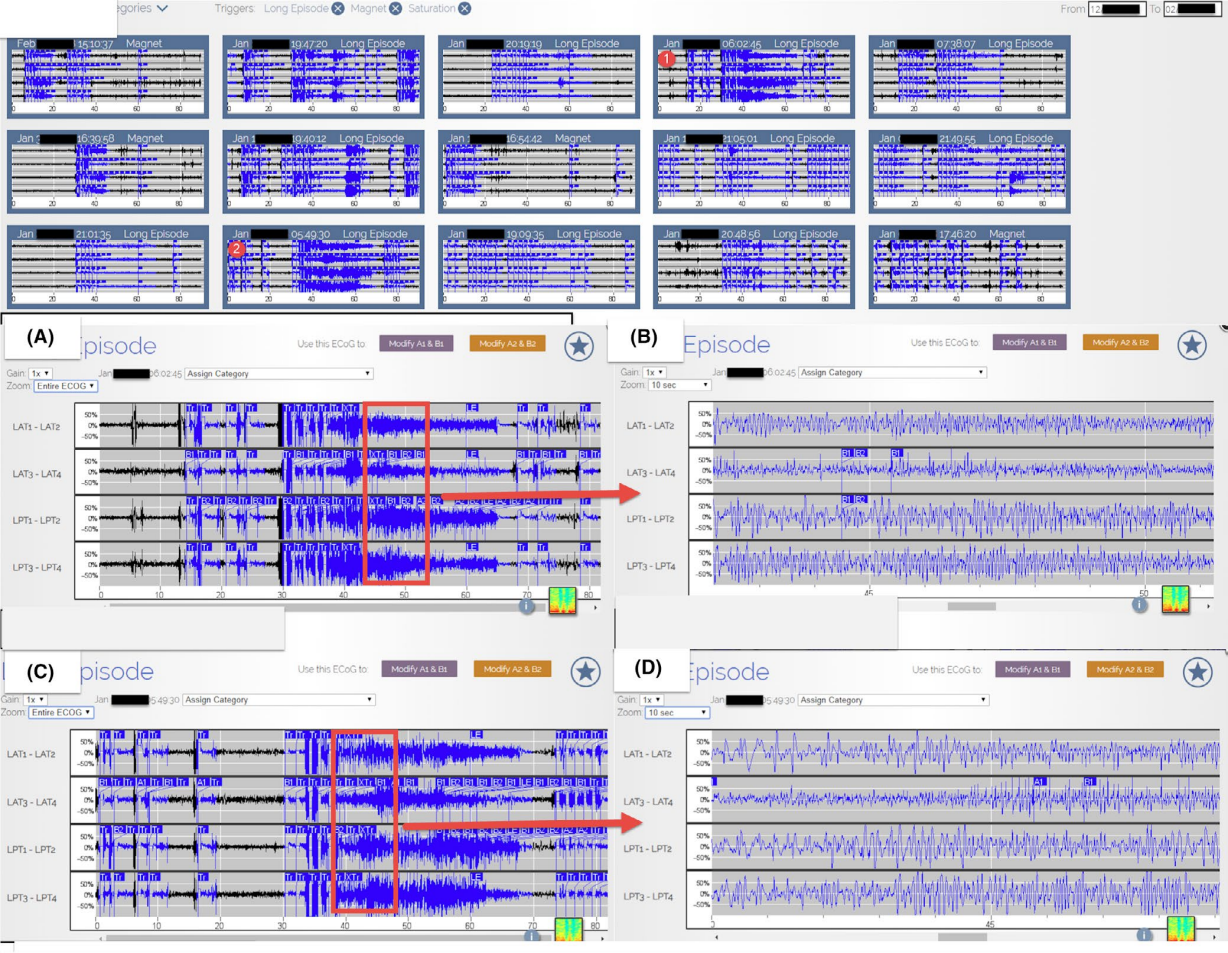
A 2‐mo period of recorded ECoG in the secure online database from 5 to 7 mo after the initiation of Lamotrigine (these ECoGs include Long Episode, Saturation, and Magnet). The Lamotrigine dosage was around 400‐500 mg QD. A is the enlargement of the red circle one, and B is the further enlargement of A and is consistent with an electrographic seizure. C is the enlargement of the red circle two, and D is the further enlargement of C and is consistent with an electrographic seizure. The number and severity of electrographic seizures decreased compared with Figure 2

**Figure 4:**
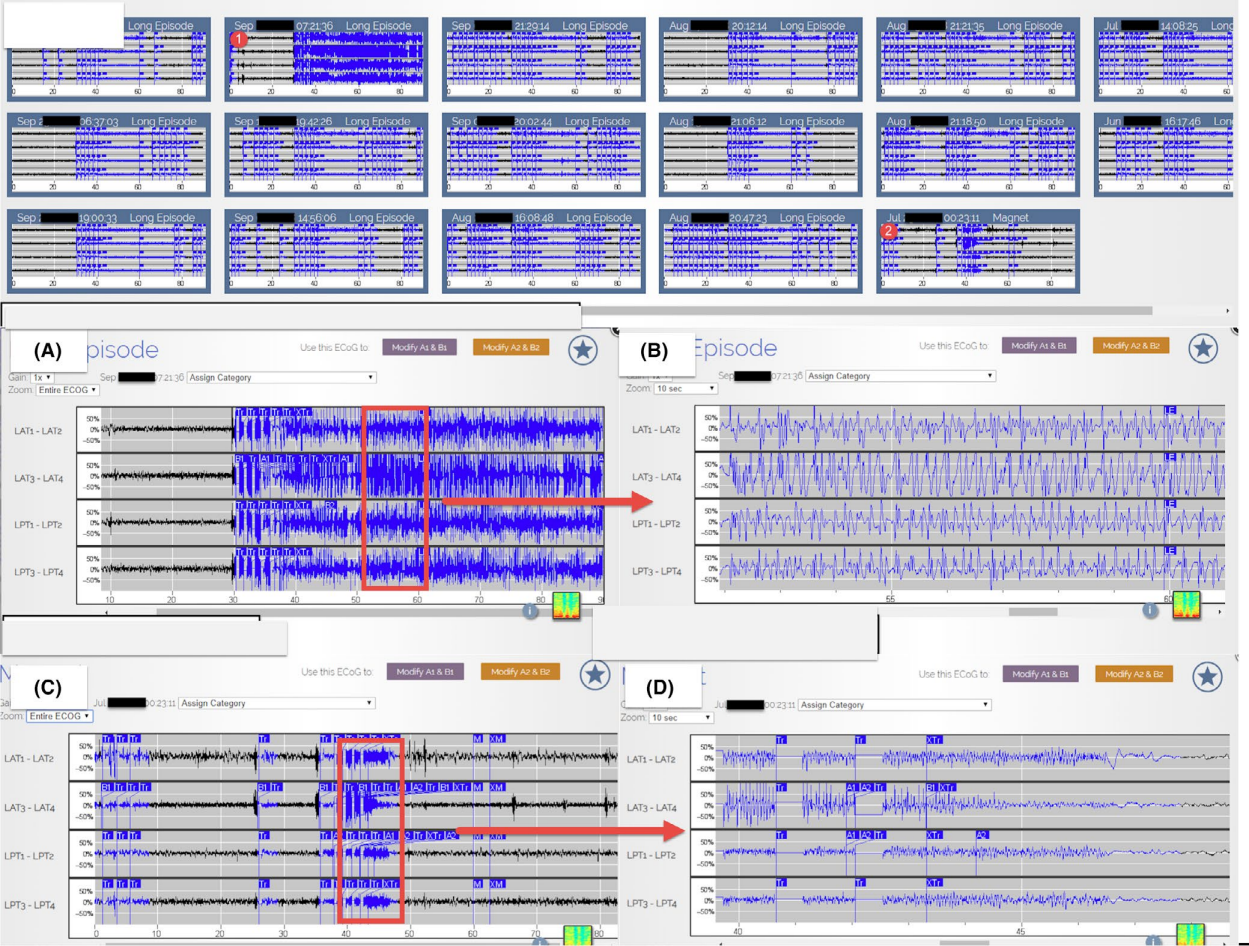
A 3‐mo period of recorded ECoG in the secure online database from 11 to 14 mo after the initiation of Lamotrigine (these ECoGs include Long Episode, Saturation, and Magnet). The Lamotrigine dosage was around 600 mg QD. A is the enlargement of the red circle one, and B is the further enlargement of A and is consistent with an electrographic seizure. C is the enlargement of the red circle two, and D is the further enlargement of C and will not be classified as an electrographic seizure because the duration of rhythmic bursts is shorter than 10 s. The number and severity of electrographic seizures decreased compared with Figure 3

**Figure 5:**
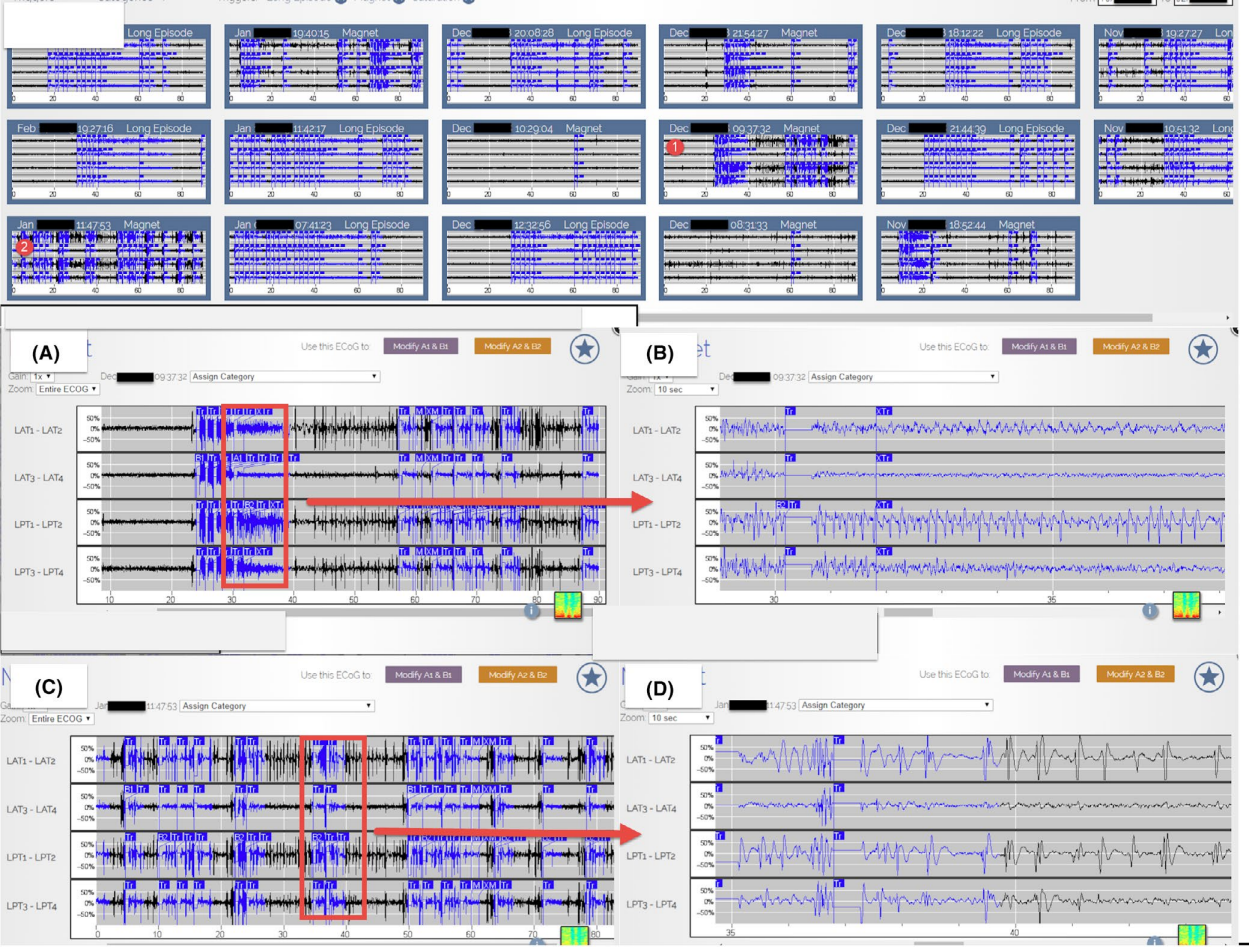
A 4‐mo period of recorded ECoG in the secure online database from 15 to 19 mo after the initiation of Lamotrigine (these ECoGs include Long Episode, Saturation, and Magnet). The Lamotrigine dosage was around 800 mg QD. A is the enlargement of the red circle one, and B is the further enlargement of A and is consistent with an electrographic seizure. C is the enlargement of the red circle two, and D is the further enlargement of 5.C and is consistent with prolonged epileptiform discharge. The number and severity of electrographic seizures decreased compared with Figure 4

Besides examining the Long Episode Count, reviewing the ECoG data of the Long Episode, Saturation, and Magnet also helps to quickly access the effectiveness of AED change (Figures 2, 3, 4, 5). Of note, the recorded ECoG only represents a portion of events, thus potentially underestimating the number of seizures due to overwriting. The extent of overwriting is inversely proportional to the frequency with which patients transfer the data (Figures 2, 3, 4, 5). Although RNS continuously monitors, it does not continuously record.^3^

## 2 CASE REPORT

This is a 31‐year‐old male who began experiencing focal seizure of deja vu lasting 10‐20 seconds about every 2‐3 weeks since the age of 14. His first secondary generalized seizure was around the age of 21. He developed focal seizures of receptive and expressive aphasia as many as 5‐10 times per day. His seizures remained intractable despite trials of many AEDs (Levetiracetam, Topiramate, Clobazam, Vigabatrin, Lacosamide, Perampanel, Lorazepam, Valium, Alprazolam, and Midazolam). Brain MRI was negative at the age of 24.

The methods to determine the site of ictal onset zone was intracranial EEG monitoring using both strip and depth electrode done in the California Pacific Medical Center (not our facility). A seizure focus was determined to be from multiple sites, including the left temporal language area. Therefore, the patient underwent multiple subpial transection surgery on two separate occasions, as well as the implantation of the RNS at the age of 27. One cortical strip and one depth lead were implanted over the left temporal in California Pacific Medical Center. The care of the patient was then transferred to our facility after the initial titrations.

We adopt the definition of “electrographic seizure” as a sustained rhythmic discharge, including repetitive spiking or spike‐and‐wave pattern faster than or equal to 2 Hz, with definite evolution in either frequency, location, or morphology. In addition, it should be clearly distinguishable from background, with at least 10 seconds in duration.^3^, ^5^ In this case, the Detection events were mostly composed of nonseizure patterns consisting of epileptiform activities or nondescript patterns. The Long Episode events were also mostly composed of nonseizure patterns, such as brief interictal epileptiform bursts, but also included electrographic seizures (<20% in this case).^1^ The events of the Saturation and Magnet sometimes consisted of electrographic seizures.

### 2.1 Responsive neurostimulation system lead configuration optimization

In the first 2 years after RNS implantation, the neurology team actively programmed the detection and stimulus parameters. The RNS's leads placement and configuration were regarded as optimal at the end of 2 years of programming stage for two reasons. First, on average, the focal seizures of speech disturbances were reduced from 5 times to once a day. However, the frequency of generalized tonic‐clonic seizure (GTCS) remained around 3‐5 times per year. Second, after reviewing the recorded ECoG data, there were samples of ECoG that demonstrated the abrupt termination of electrographic seizures after initiation of the stimulus, which supports the effectiveness of the stimulus parameters.

### 2.2 Effectiveness of antiepileptic drug optimization to achieve disabling seizure‐free quality of life

Two years after RNS implantation, the neurology team started to focus on the AED optimization because of the remaining GTCS the patient continued to experience. The RNS detection and stimulus settings were held constant to provide reliable ECoG markers in gauging the therapeutic effectiveness of AED changes. The neurology team chose to add Lamotrigine on his current antiepileptic medication regimen (Keppra 4500 mg/daily, Onfi 50 mg/daily, and Vimpat 600 mg/daily), as the patient was not previously prescribed Lamotrigine. The neurology team quickly decided that the Lamotrigine was effective because of the following: (a) The number of Long Episode count decreased (Figure 1); (b) From reviewing the ECoG data, including Long Episode, Saturation, and Magnet, the severity and amount of subclinical seizures decreased; (Figures 2, 3, 4, 5); and (c) The patient stopped having disabling seizures which were defined as GTCS and noticeable confusion after the Lamotrigine was added.

Although the frequency of disabling seizures subsided after the initiation of Lamotrigine, the neurology team continued increasing this AED in the following 1.5 year based on the ECoG data. With the stepwise increase in Lamotrigine, the severity and number of the subclinical seizures decreased further on ECoG (Figures 1, 2, 3, 4, 5). The serum Lamotrigine level increased to 12.6 μg/mL step by step. The authors believe that controlling these subclinical seizures would both prevent clinical seizures and make the patient ready for the future reduction and/or elimination of other less‐effective AEDs.

During this 1.5‐year period of drug optimization, the neurology team increased the Lamotrigine stepwise up to 800 mg daily. The patient developed adverse peak‐dose diplopia with a higher dose of Lamotrigine (greater than 800 mg) daily. The patient did not have any GTCS since the initiation of Lamotrigine. The Long Episode count showed a decreasing trend during this 1.5‐year period despite some fluctuation (Figure 1). The need for the patient to utilize Magnet Swipe also decreased. Because of the overwriting of Magnet Swipe data, the count of Magnet Swipe was not available. At the end of this stage, the patient reported that he rarely used Magnet Swipe anymore. The self‐reported focal seizures of speech disturbances reduced from once a day to once a week after the initiation of Lamotrigine. Furthermore, the severity and duration of focal seizure decreased, and the patient did not always feel the need to use Magnet Swipe.

### 2.3 Effectiveness of antiepileptic drug optimization to improve quality of life

In this stage, the neurology team started reducing other AEDs in order to improve the patient's quality of life. The parameter of Long Episode count together with reviewing the ECoG data guided the reduction in AEDs. The goals were to both keep the patient disabling seizure‐free and to minimize the adverse effects of polypharmacy by tapering less‐effective AEDs. The patient reported both a reduction in sedation and medications, specifically less‐effective AEDs. The ECoG parameters and clinical seizures did not increase with the reduction in these less‐effective AEDs.

In summary, after 2 years of active RNS programming, the neurology team held the RNS settings constant and focused on optimization of AEDs. The patient was free of disabling seizures for more than one year with the help of the ECoG (Electrocorticographic) data of RNS (Figures 1, 2, 3, 4, 5).

## 3 DISCUSSION

The chronic ambulatory ECoG data of RNS can play an important role in optimizing of AEDs. Among all parameters recorded by RNS, the recorded ECoG data (Long Episode count, Saturation, and Magnet Swipe count) are three promising parameters that can help physicians quickly decide the efficacy of AEDs and any changes made, such as dosing. Because subclinical seizures are being recorded by RNS, the physicians can have a better idea of the effectiveness of certain AEDs after any medication changes are made, including increasing or decreasing dosages, and the addition of another AED.

Because of the limited number of contacts used in the RNS system and the possibility that the recorded seizures could have reflected a spread pattern from brain regions not sampled by the electrodes rather than the ictal onset zone, the RNS leads implantation sites and the leads configurations must be optimal for the ECoG parameters to be clinically relevant. 6 In this case, this assumption is supported by the following: (a) The patient's focal seizures decreased with the RNS system being programmed 2 years after RNS implantation; (b) from reviewing the recorded ECoG data, there are samples of ECoG that demonstrated the abrupt termination of electrographic seizures after initiation of the stimulus, which supports the effectiveness of the stimulus; and (c) if the Magnet count was reliably documented, the decreasing trend of Magnet Swipe count together with the increasing trend of Long Episode count would also support the effectiveness of RNS treatment.

Adjusting antiepileptic medications is a part of the many responsibilities of most neurologists, but previously, only reports of seizures by the patient or caregiver could be used to determine whether the medication was working. Dose adjustments are also empirical and based on sparse, and often inaccurate, patient seizure counts. Using continuous data of brain activity to monitor, the response to medication changes provides near real‐time feedback and greatly facilitate medication management. In this case, the neurology team kept increasing the Lamotrigine based on the stepwise reduction in subclinical seizures recorded on the ECoG data (Figures 2, 3, 4, 5). Figure 2 displays the subclinical seizures recorded before initiation of Lamotrigine, while Figures 3, 4, 5 show the seizures after implementing different doses of Lamotrigine (2, 3, and 4 months after drug initiation). This critical information provided by this ambulatory ECoG greatly sped the optimization of the antiepileptic medication and improved the patient's quality of life.

With the current model of RNS, whenever the patient senses the focal seizures, the patient can remain compliant in swiping the magnet and transferring the ECOG data to the secure online database immediately afterward and daily. The Magnet Swipe count could potentially serve as a parameter to replace self‐reported focal seizures.

ECoG is much more sensitive than Scalp EEG. Not only can the chronic ambulatory ECoG detect seizures missed on scalp EEG, but it also detects subclinical (electrographic) seizures that the patients may not be aware of.^7^ It is reasonable to assume that controlling all seizures (subclinical and clinical) would result in better seizure management. The Long Episode count represents the summation of clinical seizures, subclinical seizures, interictal epileptiform activities, and nondescript patterns; therefore, the Long Episode count can be an overestimation for both clinical and subclinical seizures. In this case, <20% of the recorded Long Episode events met the criterion of electrographic seizures (Figures 2, 3, 4, 5). Reduction in the Long Episode count implies reduction in both the subclinical seizures and clinical seizures. The recorded ECoG samples also help to confirm the reduction in electrographic seizures (Figures 2, 3, 4, 5). It is important to note that reviewing ECoG alone can be an underestimation of the number of seizures because not all ECoG data are recorded and uploaded.

During the stage of AED optimization, the neurology team adopted the following strategies. First, choose one AED that the patient has never been prescribed before, while reviewing the recorded ECoG and examining the Long Episode count in gauging the beneficial effects of the AED change. The AED change should either be stopped if ineffective, or further increased, until the patient develops side effects or until subclinical seizures are in remission based on ECoG and the patient's self‐report. The same process could be repeated with another AED. Once the patient is free of disabling seizures for 1‐2 years, the neurology team can start the process of tapering other less‐effective AEDs to reduce the adverse effects of polypharmacy and improve the quality of life. This strategy is comparable to keeping the winners and cutting the losers of AEDs. The chronic ambulatory ECoG data of RNS serve as reliable and fast feedback for the neurology team to optimize the AEDs.

## 4 CONCLUSION

The RNS system is regarded as one adjunctive treatment for medically intractable epilepsy 8. The case demonstrates that the chronic ambulatory ECoG data of the RNS System could speed the process of assessing the clinical effectiveness of AEDs by guiding AED choice and appropriate dosing to achieve subsidence of disabling seizures. Besides the recorded ECoG, the Long Episode count serves a good estimate of the summation (number of seizures) of clinical and subclinical seizures. The RNS system together with AED optimization strategy based on the ECoG parameters provided by RNS could open new ways to ultimately achieve subsidence of disabling seizures and to minimize the adverse effects of polypharmacy by tapering less‐effective AEDs, thus improving the patient's quality of life.

## CONFLICT OF INTEREST

The authors declare no conflicts of interest.

## AUTHOR CONTRIBUTIONS

ET: prepared this manuscript. YYP: was the neurologist for this patient.
